# Validation of Sensor-Based Food Intake Detection by Multicamera Video Observation in an Unconstrained Environment

**DOI:** 10.3390/nu11030609

**Published:** 2019-03-13

**Authors:** Muhammad Farooq, Abul Doulah, Jason Parton, Megan A. McCrory, Janine A. Higgins, Edward Sazonov

**Affiliations:** 1 Department of Electrical and Computer Engineering, University of Alabama, Tuscaloosa, AL 35487, USA; mfarooq@crimson.ua.edu (M.F.); adoulah@crimson.ua.edu (A.D.); 2 Department of Information Systems, Statistics, and Management Sciences, Culverhouse College of Business, University of Alabama, Tuscaloosa, AL 35487, USA; jmparton@cba.ua.edu; 3 Department of Health Sciences, Boston University, Boston, MA 02215, USA; mamccr@bu.edu; 4 Department of Pediatrics, University of Colorado Anschutz Medical Campus Denver, Aurora, CO 80045, USA; janine.higgins@childrenscolorado.org

**Keywords:** obesity, dietary assessment, chewing detection, AIM, neural networks, food intake detection, video annotation, sensor validation

## Abstract

Video observations have been widely used for providing ground truth for wearable systems for monitoring food intake in controlled laboratory conditions; however, video observation requires participants be confined to a defined space. The purpose of this analysis was to test an alternative approach for establishing activity types and food intake bouts in a relatively unconstrained environment. The accuracy of a wearable system for assessing food intake was compared with that from video observation, and inter-rater reliability of annotation was also evaluated. Forty participants were enrolled. Multiple participants were simultaneously monitored in a 4-bedroom apartment using six cameras for three days each. Participants could leave the apartment overnight and for short periods of time during the day, during which time monitoring did not take place. A wearable system (Automatic Ingestion Monitor, AIM) was used to detect and monitor participants’ food intake at a resolution of 30 s using a neural network classifier. Two different food intake detection models were tested, one trained on the data from an earlier study and the other on current study data using leave-one-out cross validation. Three trained human raters annotated the videos for major activities of daily living including eating, drinking, resting, walking, and talking. They further annotated individual bites and chewing bouts for each food intake bout. Results for inter-rater reliability showed that, for activity annotation, the raters achieved an average (±standard deviation (STD)) kappa value of 0.74 (±0.02) and for food intake annotation the average kappa (Light’s kappa) of 0.82 (±0.04). Validity results showed that AIM food intake detection matched human video-annotated food intake with a kappa of 0.77 (±0.10) and 0.78 (±0.12) for activity annotation and for food intake bout annotation, respectively. Results of one-way ANOVA suggest that there are no statistically significant differences among the average eating duration estimated from raters’ annotations and AIM predictions (*p*-value = 0.19). These results suggest that the AIM provides accuracy comparable to video observation and may be used to reliably detect food intake in multi-day observational studies.

## 1. Introduction

Monitoring and assessment of dietary intake and eating behavior is essential for studying and understanding the factors contributing to obesity and over-weight [1,2]. Traditional approaches of dietary intake assessment utilize self-report methodologies such as 24 h dietary recall [3], food frequency questionnaires [4], and electronic devices for record keeping such as personal data assistants and smart-phones [5]. However, these methods rely heavily on participants’ input which results in participant burden and may also result in inaccurate data [6,7]. Over the past decade or so, several automatic food intake detection approaches have been proposed to address the problematic issues associated with self-report by employing different sensing modalities, such as acoustic [8], piezoelectric (e.g., strain gauge) [9,10,11] and inertial (e.g., accelerometer [11,12]) sensors. Sensor-based approaches require validation for data collection, signal processing, and pattern recognition methods. Many sensors have been validated in laboratory studies; however, validation in unconstrained, free-living or pseudo-free-living environments is required for realistic assessment of sensor performance [13]. For validation, having a robust and objective ground truth metric is essential. Three different methodologies have been widely used for establishment of ground truth data for food intake detection including (1) external observer; (2) push-button by the participant, and (3) video observations of individuals.

External observers have been used extensively to establish ground truth in previous studies. For example, several studies using wearable sensors such as ear-pad microphone [14], acoustic sensor around the neck [15,16] have employed external observers to monitor subjects and manually annotate the collected sensor data. Methods relying on external observers can be labor intensive and may not be accurate for marking the start and end of eating activity as the observers themselves are not involved in the eating activity and mostly rely on visual observation. Another popular approach for ground truth collection is the annotation by the subjects themselves using either pushbutton or mobile apps and have been used in conjunction with a wide variety of sensors such as piezoelectric strain sensor [10,17,18], smart eye-glasses [11,19], and acoustic sensors [20]. The use of push-button by the participants can provide comparatively accurate start and end times of eating activity and therefore could potentially be used for accurate assessment of the developed sensors and related signal processing and pattern recognition methodologies. However, the presence of a push-button can impact the way people would normally eat and interact with their environment (i.e., one hand is always busy with the pushbutton) and could also potentially increase participant burden as well as result in inaccurate labels if the participant is distracted. The accuracy of push-button annotation by participants is also dependent on the participants pushing the button at the correct time (i.e., at the actual start and end times of eating). Therefore, there is a need for assessment methods which do not rely on users.

Another approach for establishing the ground truth data is through video observation of individuals and does not rely on the users. This approach can potentially be used in conjunction with any wearable sensor for monitoring food intake such as chewing and swallowing monitoring systems (piezoelectric strain sensor, swallowing microphones, and electroglottography) [8,21,22,23,24,25,26], and wrist monitoring systems for tracking bites (for example MEMS gyroscope based system for tracking wrist movements [27], accelerometer present in smart-watches [8]). Video-based annotation methodology has also been utilized in the studies [25,26] for monitoring the feeding behavior of infants in laboratory conditions. A common theme among all the studies which relied on the video observation is the use of a single camera fixated on the participant. This restricts participants to a small defined space, e.g., a dining table, and fails to capture daily activities of the participants. Using a single camera also limits the number of participants that are generally recruited for a study session and usually needs one camera per participant. Video based observations are sensitive to the quality of images/videos taken, orientation of the camera, closeness of the camera to the participant, etc. Another problem associated with video observation is that the results are subjective and dependent on inter- and intra-rater reliability of the human annotators. Therefore, multicamera systems are required which can capture a wide variety of activities performed by the individuals and do not restrict the movements of participants to a designated table/space. At the same time, it is essential to evaluate the inter- and intra-rater reliabilities of the annotation procedure to account for subjectivity of the annotators.

This paper presents results of a study in which multiple participants were monitored simultaneously in a multiroom (4-bedroom) apartment with six cameras installed in different locations. Each participant was wearing a multisensor system called Automatic Ingestion Monitor (AIM [10]) for automatic monitoring of food intake related events. The study was conducted with multiple goals: (1) to establish the reliability of video observations for monitoring food intake bouts using wearable sensors in a pseudo-free-living testing environment; and (2) establish the accuracy of the sensor-based food intake predictions with respect to video observation and evaluate if the AIM sensors can be used as a replacement for video observation in unconstrained environments.

## 2. Materials and Methods

### 2.1. Data Collection Protocol

Forty (20 male and 20 female) healthy participants were recruited (aged 24.5 ± 3.4 years; Body Mass Index (BMI) 26.1 ± 5.2 kg/m^2^; Mean ± STD). Participants were recruited by advertisements placed around the University of Alabama, Tuscaloosa area and in the University newsletter. Individuals were screened for medical conditions which would impact normal chewing. Those with a history of eating disorders, food allergies or sensitivities, or other conditions which resulted in avoidance of consumption of a wide range of foods (e.g., gluten intolerance, peanut allergy) were excluded from the study. The study protocol was approved by the University of Alabama Institutional Review Board and all individuals provided informed consent before participation in the study.

### 2.2. Sensor System

Participants were asked to wear a multisensor system AIM (v1.0) [10] comprised of three components: a hand gesture sensor worn on the dominant hand, a piezoelectric strain sensor (LDT0-028K from Measurement Specialties Inc., Hampton, VA, USA) placed on the jaw using medical adhesive, and a data collection module worn around the neck using a lanyard. The hand gesture sensor had an RF transmitter (data sampled at 10 Hz), whereas the data collection module had an RF receiver, and both acted together as proximity sensor to detect characteristic hand to mouth (potential bite) gestures. The data collection module also had preconditioning and signal processing circuitry for the jaw motion sensor (sampled at 1000 Hz). It also included a triaxial accelerometer (ADXL335 from Analog Devices, Norwood, MA, USA) for detecting body acceleration (sampled at 100 Hz). Data from the accelerometer was used for determining physical activity levels. Each participant was also provided with an Android smartphone with a dedicated app to collect data. Data from the data collection module were wirelessly transmitted to the phone via RN-42 Bluetooth module with serial port profile. Details about the sensor system used in this study can be found in [10].

### 2.3. Experimental Protocol

**Figure 1:**
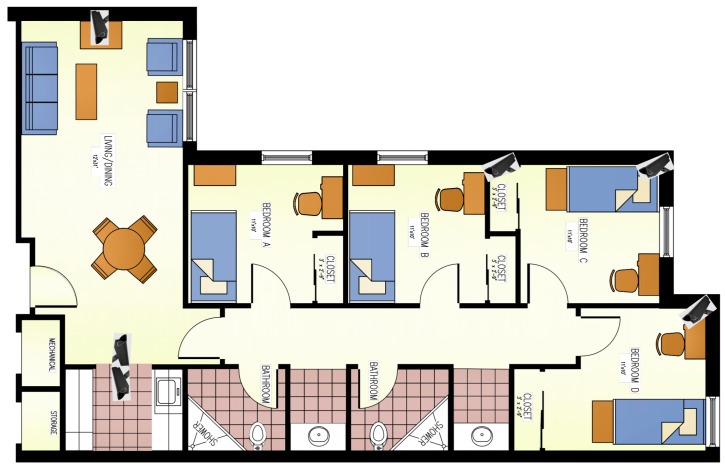
Floorplan of the apartment and placement of the six cameras in the apartment. Cameras were placed such that the area of the coverage is maximized.

The observational facility was a 4-bedroom, 3-bathroom apartment with a common living area and kitchen. One of the bedrooms was used by the research staff and therefore, was blocked from access to the participants. Each bedroom had a bed, a study chair and desk; while the living area had a sofa, chairs, dining table, a TV with a game console, and a stationary cycle. The kitchen shelves and refrigerator were fully stocked with daily eating supplies and a variety of different foods (189 items) and the supplies were replenished on regular basis to ensure that none of the items were ever out of stock. A daily inventory was kept of the items consumed. The facility was instrumented with 6 motion-sensitive cameras to capture all the activities performed by the participants. Cameras used in the study were GW-2061IP (GW Security, Inc., El Monte, CA, USA), which provided video recording at fully HD resolution (1080p). The locations of the cameras in the apartment are shown in the Figure 1. Bathrooms were not monitored due to privacy concerns. Participants were asked to eat only in rooms that were equipped with cameras.

Each participant completed the study over three days which were scheduled based on their availability and had an interval of at least three days in between each test day. On any given day, there were no more than three participants in the observational facility. This facilitated interactions among the participants throughout the day, including during meals. On each of the study days, participants reported to the observation facility between 7:00–8:00 a.m. and participated in the experiment until 8:00 p.m. Participants were trained on how to place the piezoelectric strain sensor on the jaw and then the participants self-applied the sensor each study day. For all eating occasions, participants had the option of either eating from the food items available in the apartment’s kitchen or to get food on the UA campus at one of the three cafeterias or a food court with multiple fast food vendors. Participants could eat at any time of their choosing, as many times as they wanted, as much as they wanted. They could leave the facility for short periods of time during which they were not monitored. Research assistants kept a record of these times and they were subsequently excluded from the analysis. Upon completion of each study day, participants removed the sensor system and were free to leave.

### 2.4. Annotation Procedure

**Figure 2:**
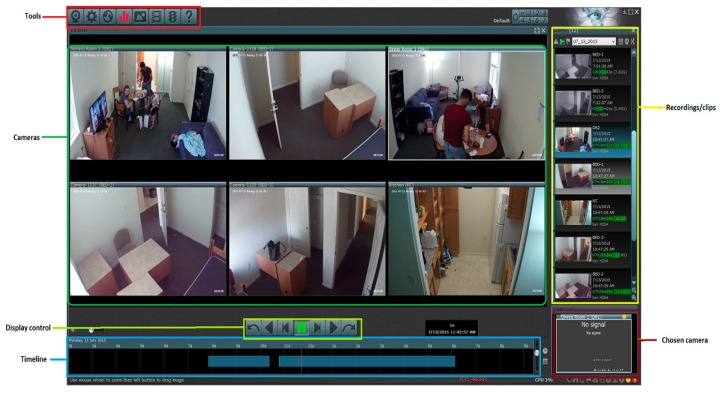
A snapshot of the software used for video observation and annotation. The annotator can view all six cameras simultaneously and can mark start and end of different activities.

To identify the ground truth for each participant’s activities, the video recordings were manually annotated by three trained human raters (training described below). The annotation process included two stages—(1) activity annotation and (2) food intake bout annotation. In this case, a food intake bout is defined as a single sitting of eating which involves several bites and chewing bouts and may or may not involve liquid intake. This could be a full meal or a small snack. Figure 2 shows an example of the video screenshot of all six cameras that the raters could see and annotate simultaneously. The activity annotation consisted of identification of six categories: eating food intake bout boundaries, drinking, physically active, physically sedentary, talking, and out of view. Brief definitions of these categories of activities are provided in Table 1a. Some constraints were placed during activity annotation as shown in Table 1b. Out-of-view segments of the videos were not included in the analysis. Start and end time of each activity were recorded.

**Figure 3:**
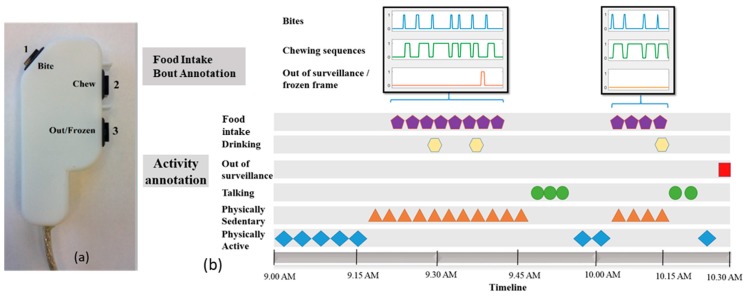
(**a**) The three button systems for annotating the videos of food intake both act activity level as well as meal level; (**b**) Example of the annotation procedure both at the activity and food intake bout level.

After the completion of activity annotation, each food intake bout was further annotated with finer details of individual bites and chewing sequences. Food intake annotations were performed by using a 3-button system and a custom-built software. The 3-button system is shown in Figure 3a, in which button-1 and button-2 were used to indicate bite and chewing events respectively. Additionally, a third button was employed to record potential out of view/frozen video frames. Brief definitions of these categories of events in food intake bout annotation are provided in Table 2. Figure 3b shows an example of the annotation procedure both at activity level and food intake level. For a typical food intake bout, a bite is followed by a sequence of chews and one or more swallows. Swallowing events were difficult to see in the video recordings; therefore, they were not annotated. There were cases where video frames were lost and the transition among the frames was not smooth. This manifested as frozen image frames. Timestamps corresponding to these frames was noted and they were not included in the analysis.

### 2.5. Training of Human Anotators

All the raters were trained before conducting annotation on the full dataset. During training, the raters were provided with specific instructions and supervised by an expert. As a part of activity annotation training, the raters annotated 10 h of video recording. The full day video was played at a high playback speed (×8) and raters were instructed to pause the video at times when any of the six activities took place. To improve annotation, the raters used rewinding and forwarding of the frames when necessary to identify the start and end times of any category. In addition, raters also used time-stamp information from the research assistant records along with the video observations to annotate videos. Since multiple participants could appear in the camera view, the raters were instructed to complete annotation for one participant at a time and to ignore the other participants who appeared in the video.

Like the activity annotation, raters were given training on use of the 3-button system and custom-built program to annotate food intake bouts. In the training, the raters identified every bite and chewing sequence that took place within a food intake bout. They were instructed to press button-1 once and release immediately each time the participant took a bite. The chewing button was pressed for each entire chewing sequence. The 3rd button was pressed and held for as long as the participant was out of view and for frozen video frames. This process continued until the participant finished the eating event.

### 2.6. Sensor Signal Processing and Pattern Recognition

One of the goals of the study was to establish the reliability of food intake detection by AIM with respect to the video observations. The same technique can be used for validation of any other sensor for food intake detection. For the validation of the AIM, annotated data was used as reference. Two models for food intake detection were tested. The first model was obtained on an independent dataset trained in a previous study which consisted of a data from 12 participants who wore the AIM device for 24 h [10]. Those participants didn’t participate in the current study. Data from the current study were used for testing purposes only. Food intake was detected as 30-s segments labeled as food intake or non-food intake. The data preprocessing and feature computation algorithms were applied to the sensor signals as presented in [10] to ensure that models trained in [10] could be used in this study. The second model utilized the neural network architecture presented in [10], but was trained and validated on data collected during the present study. In this case, a leave-one-participant out cross validation scheme was used, where data from one participant (all days) were used for testing and data collected from the rest of the participants were used for training of the neural networks.

### 2.7. Statistical Analysis

Statistical comparison was performed to measure the agreement among the raters, and among the video annotation and the AIM-detected food intake. For computing agreement, Cohen’s kappa (κ) based inter-rater reliability testing was computed for both activity and food intake bout annotation. The kappa is represented by the following formula:

(1) $$\kappa =\;\frac{\mathrm{Prob}\left(a\right)-\mathrm{Prob}\left(e\right)}{1-\;\mathrm{Prob}\left(e\right)}$$

where Prob(a) and Prob(e) represent the probability of observed agreement and expected agreement respectively. The κ can range from −1 to +1, where values κ ≤ 0 indicate no agreement, 0.60 < κ ≤ 0.80 indicate satisfactory agreement and κ > 0.80 represent almost perfect agreement.

The inter-rater reliability of the marking of food intake bout boundaries (in the case of activity annotation) and chewing sequences (in the case of food intake annotation) was also evaluated. To evaluate the performance of activity annotation, 1 day of 10 h of video was annotated by each of the three raters after they were trained. For food intake annotation, 10 meals were annotated by each of the three raters.

The following comparisons were performed. To examine inter-rater reliability among the raters, kappa statistics between the three raters were computed and then averaged to obtain Light’s kappa. Light’s kappa indicates the agreement among the raters when the same day data is annotated by multiple raters. For performance evaluation of the AIM, Light’s kappa was used to measure the agreement between the prediction by the AIM and a human rater. For completion, we have also reported the F1-score; which is widely used for performance evaluation of machine learning models. The F1-score is the weighted average of recall and precision. Recall indicates the true positive rate whereas the precision indicates the positive predictive values of the classifier.

Further, a comparison among the average eating duration estimated using the activity level annotation and food intake bout level annotation of the video and AIM prediction is also provided. One-way analysis of variance (ANOVA) was performed with a null hypothesis that average eating duration from all three methods are not statistically different with a *p*-value of 0.05.

## 3. Results

For marking food intake events’ boundaries in activity annotation, Light’s kappa (agreement among the raters) was 0.74. For marking chew sequences in food intake bout annotation, Light’s kappa was 0.82. Results of the AIM prediction in comparison to the video annotations are given in Table 3 and Table 4. Both activity and meal level predictions from the AIM achieved satisfactory agreement with video annotation (Cohen’s kappa of 0.77 and 0.76 respectively, for models trained on the present study dataset). Table 3 also shows the F1-scores achieved by the classifier for both predicting the activity- and meal-level annotations. Table 4 shows the results of AIM prediction when AIM models were trained on the independent dataset from our previous study.

Table 5 shows statistics on the durations of the experiments (from start to end), eating duration marked by the activity level food intake bout annotation, as well as the eating durations predicted by AIM. One-way ANOVA shows that there are no statistically significant differences (*p*-value 0.19 > 0.05) among the average eating durations (over a day) among activity level annotation, food intake bout level annotation, and the AIM-predicted eating durations.

## 4. Discussion

The presented study investigated several issues related to evaluation of wearable sensors for food intake detection in pseudo-free-living environments. Multicamera video observation was used as the gold standard in detection of food intake, instead of relying on pushbuttons which has limitations [10]. As previous research has shown [13], eating behavior varies significantly between strictly controlled laboratory conditions and less restrictive, semi-constrained, or free-living environments. Use of video observation may be a useful tool in establishing the ground truth under the latter conditions.

The use of video-based observation as a means of AIM sensor validation facilitated low participant burden as participants were not required to record their food intake events. Such an approach has multiple advantages. First, not relying on participants to self-report their intake could potentially reduce inaccurate data collection. In addition, presence of multiple cameras did not restrict participants to a confined eating space and they could eat anywhere in the four-bedroom apartment. This approach may have helped the participants mimic their usual daily eating habits, which is desirable in studies of diet and health outcomes.

The inter-rater reliability results for the annotation showed some variability among the raters’ perception of eating and not eating. Kappa values of 0.74 (74% agreement) for activity annotation and 0.82 (82% agreement) for food intake bout annotation is good, but not perfect. Although video-based food intake observations have been extensively explored for monitoring in very constrained, laboratory studies, their use in free-living conditions to provide ground truth for wearable sensors may be less reliable as indicated by the kappa for inter-rater reliability metrics. A possible alternative to video-based observation is to use wearable sensors such as the AIM for continuous non-invasive monitoring of eating behavior. Wearable sensors can potentially provide more objective monitoring compared with video-based observations.

Two separate AIM prediction models were tested in this study, and both were compared to video annotation. One model was trained on an independent dataset and the second model was trained on the data collected in the present study. Both models produced results comparable to video annotation, with the first (independent) model resulting in kappa values of 0.74 for activity and 0.71 for meal level annotation. As expected, the recognition model trained on the present dataset had relatively higher agreement (0.77 and 0.76 agreement with raters for activity and food intake bout level annotation respectively) compared to the AIM models trained on independent data. In comparison, inter-rater agreement among raters was 0.74 and 0.82 for activity and food intake bout level, respectively.

One of the possible factors contributing to the strong but not perfect agreement between the AIM detection and the video annotation is the granularity of the epoch size (30 s) used for sensor data processing. This granularity was greatly improved in more recent iterations of the AIM devices [19,28], which were not available at the time of the present experiment. Another source of error is the discrepancy in the observer’s ratings which, in turn, affected the fidelity of the AIM predictions. The moderate agreement among raters for video annotation and hence the AIM performance may be attributed to several factors. In some cases, very short snacking events such as eating a small piece of candy may have been missed by the raters. However, such short events were likely captured by the AIM since the AIM is continuously monitoring food intake. Disagreement between the video observation and the AIM could also potentially be explained by constraint # 2 (Table 1b) imposed on the annotation where it was decided that the eating and talking could not be annotated simultaneously. This was because when participants were sitting far from the camera, raters had to zoom in to view the participant, making the view granular and blurry. Raters faced difficulties distinguishing between food intake or talking in such blurred frames. While this could have potentially introduced inaccuracies in the annotation, the AIM would still be likely to capture chewing events during talking if chewing lasts longer than 15 s in a 30-s epoch. A previous study showed that the AIM is able to detect chewing while talking [19]. Another major limitation of identifying ground truth through video observation was the confidence (or lack of thereof) of human raters in their correct identification of the activity shown on video. Many human activities are complex and do not fall easily into predefined categories. Similarly, the raters’ expectancy (see what one wants to see) may also have contributed to error. In previous studies ([10,22]), the AIM was to able to distinguish between eating and other activities such as talking and walking etc. and therefore is potentially less prone to the difficulties encountered in video-based observation.

The average experiment duration for all participants was about 10 h out of which about 1 h (66.1 min) (based on the activity level annotation) was spent on eating related activities. The estimated average eating time based on the food intake bout level annotation was 37.1 min, whereas the average estimated eating time based on AIM predictions was 49.4 min. Higher AIM predicted eating durations can be explained by the possibility of raters not being able to mark some chewing events due to occlusion or hard to distinguish eating vs. other activities such as talking. Considering the difficulties in annotating fine level chewing, AIM predicted durations are expected to be more than the fine level chewing (food intake) and less than the activity level eating annotations. However, the one-way ANOVA showed that the differences among the average eating duration are not statistically significant. This shows that estimated eating duration from AIM can provide a good estimate of actual eating duration.

A previous shorter study in free-living conditions that used a push button ground truth reference achieved an average F1-score of 89% when tested on 12 participants using the AIM for 24 h [10] compared to an F1-score of 80% for the present study. However, we would expect similar performance provided more accurate ground truth signal is present. Results of the present study showed that the AIM can provide a reliable prediction of food intake and can potentially be used in place of direct video observations which is labor-intensive and prone to error. Sensors used for passive and automatic detection and identification of food intake have previously been shown to be able to accurately estimate chew counts and chewing rate [22]. Li et al. has shown that increasing number of chews per bite in both obese and healthy participant reduced overall food intake [29]. AIM-like devices can be used for providing near real-time feedback on chewing behavior of individuals and have shown to modify eating behavior to reduce energy intake in a single meal [30]. Similar sensors have been shown to be able to estimate mass of intake only by monitoring chewing behavior [14,31]. While the AIM can accurately detect eating events and can provide information about chewing behavior, in its current form, it does not have the ability to recognize the type of food being consumed which is critical for monitoring caloric intake. Further, integration of computer vision techniques for identification of food type will greatly improve the practical usage of the AIM and similar wearable systems. The present study, together with previous studies in this area, show that wearable systems can be used for not only detecting food intake but also providing other valuable information about eating behavior including quantification of eating rate, duration, and frequency.

## 5. Conclusions

Human raters achieved an average kappa value of 0.74 and 0.82 for higher level activity annotation and for finer food intake bout level annotation of eating occasions. The AIM predictions were compared with the human raters and achieved a kappa value of 0.8 for detection of food intake. AIM-predicted average eating durations were close to video annotated eating durations. These results indicate that the AIM can potentially be used in studies of food intake in unrestricted environments and provide performance like video annotation without the limitations associated with video annotation.

## Figures and Tables

**Table 1:** (**a**) Definitions of categories for activity annotation; (**b**) Constraints placed on activity annotation.

(**a**)	
**Category**	**Definition**
Food Intake	Participant was consuming solid food items or solid foods combined with liquids. Eating involved taking bites, chewing, and swallowing of the foods.
Drinking	Participant was consuming just liquids, no bite/chewing were involved.
Physically active	Participant were moving
Physically sedentary	Participant was not in motion, including sitting on the couch/chair, working on the computer or laying down on the bed etc.
Talking	Participant was talking to other participants or talking on the phone.
Out of view	Participant was not in the view of any of the 6 cameras
(**b**)	
**Constraints**	**Definition**
1	Participant cannot be physically active and sedentary at the same time.
2	Participant cannot be eating/drinking and talking at the same time.
3	Participant cannot be out of surveillance and physically active at the same time with the exception that when the participant was out with the research assistant getting the food, that was considered as physically active.
4	Restroom use was considered as an out of surveillance category.

**Table 2:** Definitions of categories for food intake bout annotation.

Category	Definition
Bite	The moment the participant placed the food into mouth and bit down.
Chewing bout	Tracking the jaw movement of the participant immediately after bite until swallowing the food.
Out of view/frozen frame	Frozen video frames or out of camera view (i.e., the participant was not in the selected camera)

**Table 3:** Comparison of food intake detection between video based human annotation and AIM predictions based on leave-one-out cross validation.

	Kappa		F1-Score	
	Activity Level	Food Intake Bout level	Activity Level	Food Intake Bout Level
Mean	0.77	0.76	0.8	0.78
STD	0.1	0.12	0.1	0.12

**Table 4:** Comparison of food intake detection between video based human annotation and AIM predictions based on the model from an earlier study [10].

	Kappa		F1-Score	
	Activity Level	Food Intake Bout level	Activity Level	Food Intake Bout Level
Mean	0.74	0.71	0.77	0.74
STD	0.14	0.11	0.12	0.09

**Table 5:** Statistics on Duration of Experiments, Activity, and Food intake bout level eating duration and AIM predicted eating duration. All durations are in minutes.

	Total Duration	Activity Level (Video)	Food Intake Bout Level (Video)	AIM Predicted
Mean	608.6	66.3	37.1	49.4
STD	63.5	30.4	13.4	13.7
25%	589.0	45.9	27.3	40.1
50%	619.3	55.3	35.3	48.3
75%	647.4	78.1	43.4	57.5
